# Supplementary figures and images for: Neighborhood characteristics and HIV treatment outcomes: A scoping review
Source: PLOS Glob Public Health. 2024 Feb 13;4(2):e0002870. doi: 10.1371/journal.pgph.0002870 (PMC10863897; doi:10.1371/journal.pgph.0002870)

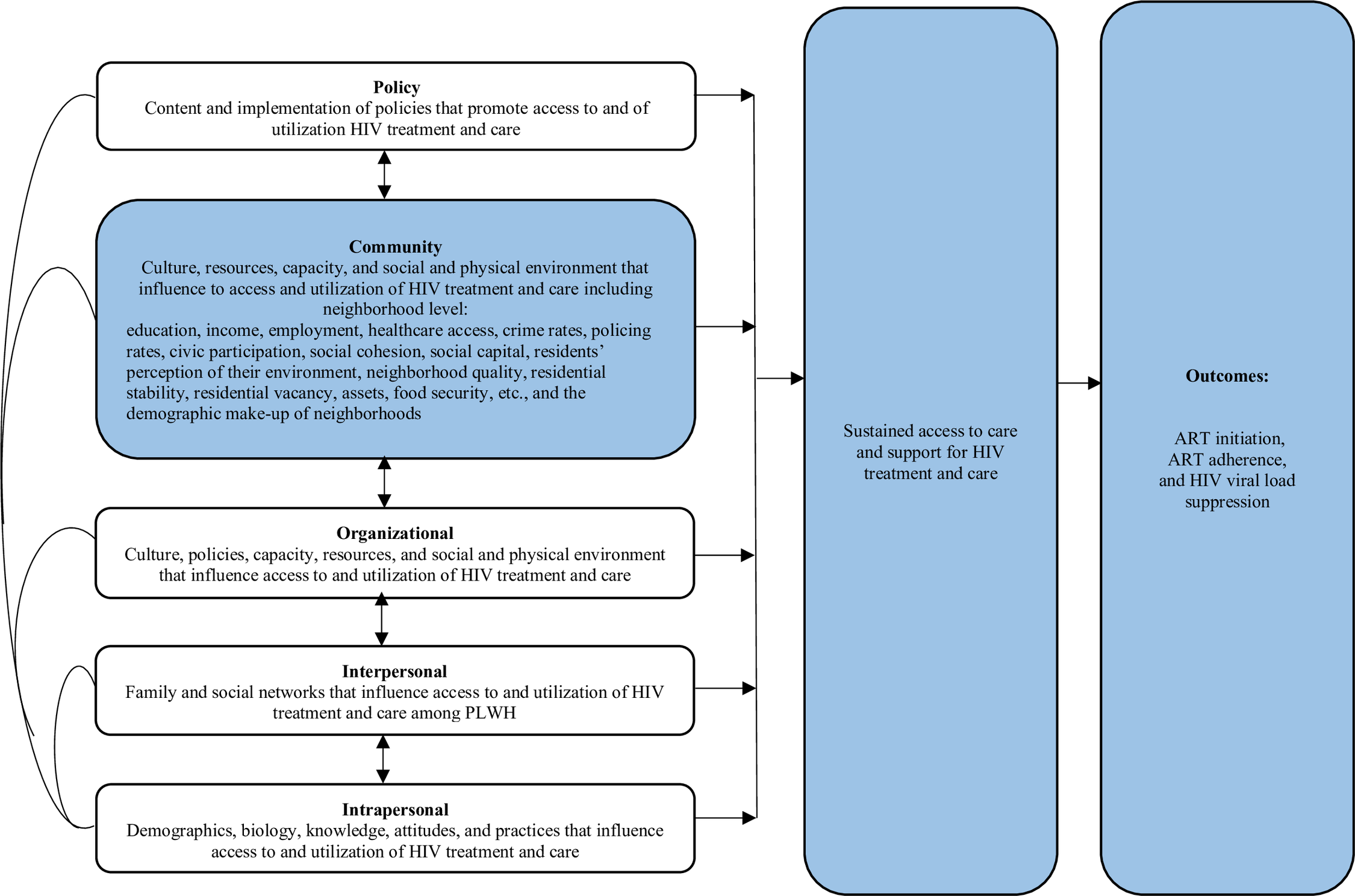

Supplement: S1 File — (TIF) [file pgph.0002870.s001.tif]
