# Supplementary material for: Neighborhood characteristics and HIV treatment outcomes: A scoping review
Source: PLOS Glob Public Health. 2024 Feb 13;4(2):e0002870. doi: 10.1371/journal.pgph.0002870 (PMC10863897; doi:10.1371/journal.pgph.0002870)
Supplement: Attachment — Submitted filename: LKimaru_Rebuttal Letter.docx [file pgph.0002870.s003.docx]

University of Arizona

Mel and Enid Zuckerman College of Public Health

Department of Health Promotions Science

1295 N Martin Ave, Tucson, AZ 85724

P: +1 6024751517

E: [lkimaru@arizona.edu](mailto:lkimaru@arizona.edu)

The Staff Editor

Jianhong Zhou

PLOS Global Public Health

14 June 2023

**Re: Manuscript ID PGPH-D-23-00460 titled "** **Neighborhood characteristics and HIV treatment outcomes: A scoping review"**

I would like to thank you and the reviewers for the time and effort spent in evaluating our manuscript. We also thank you for considering our manuscript, and the opportunity to revise following peer review.

We are appreciative of the vital comments raised by the reviewers and provide a revised manuscript. We have carefully addressed each of the concerns raised by the reviewers and made necessary revisions to the manuscript. Please find below a point-by-point response to the comments.

| Reviewer’s Comment | Author’s Response | Page(s)- p  Line(s)- L |
| --- | --- | --- |
| **Reviewer #1 - Abstract:**   1) The first sentence is very dismissive of people living with HIV. In this era, people are living with HIV for many years and there is much literature detailing the unique health-related issues PLWH face. I recommend the first sentence be appropriately revised so as not to turn off readers right at the outset. 2) The justification for why this paper is important is not compelling in the abstract – I recommend a more honed introduction section. | 1) The first sentence has been modified to focus on recognizing the challenges faced by people living with HIV.  2) The justification for this paper has been modified to focus on the central issue of the review which is assessing neighborhood characteristics’ role as community-level factors that impact HIV treatment outcomes due to the influence they may have on individual-level interventions. | 1) p.2, L:22  2) p.2 L: 23-26 |
| **Reviewer #1 - Background:**   3) The first few paragraphs read as if they was written decades ago. The benefits of ART and the HIV care cascade are well-established, and I encourage the authors to quickly get to the issue of neighborhood factors given this is the focus of their paper. 4) I would not focus so much on where neighborhood-level data is obtained, but instead delve more deeply into issues of why neighborhood characteristics are important (separate from individual-level factors), and opportunities they bring for intervention. | 3)The first and second paragraphs of the original draft have been consolidated to give a quick review of the topic of focus in one paragraph.  4) The third and fourth paragraphs of the original draft have been combined into one paragraph to provide a succinct explanation of why neighborhood characteristics are important (separate from the individual factors) and the opportunities they bring. I have also removed details of where the neighborhood-level data is obtained. | 3-4) p.3-4 L:48-91 |
| **Reviewer #1 - Methods:**   5) There is no justification why particular neighborhood-level indicators were deemed as important by the authors. For example, “physical decay” was included, but not “residential stability”. The choices they made are fine, but need to be justified. | 5) Justification included “We used general search terms to capture indicators that are commonly used in the literature to describe neighborhood characteristics. However, indicators that were less common such as (eg. physical decay, physical or social disorder) we included those terms explicitly as part of the search strategy.” | 5) p.5 Lines 111-114 |
| **Reviewer #1 - Results:**   6) The constructs of “SES” or “Neighborhood Deprivation” are likely treated differently in each study given the lack of clarity in definition of these constructs. This needs to be better differentiated in the text. 7) There is discussion of “racial/ethnic group density” that is not nuanced or carefully interpreted. Given the discourse surrounding how race and ethnicity should now be talked about, this text needs to be better developed. | 6)First, the results have been re-ordered to have concepts with similar neighborhood characteristics follow each other to improve the flow and differentiation of the concepts. The order is as follows: SES (range of resources), Deprivation (insufficient resources), Disadvantage (living below poverty line), Composition (racial/ethnic densities) and Interaction (belonging), urban/rural status (overall population densities and urbanization), and finally disorder (safety and order). Each of these concept sections have been detailed with definition or clarification of how they differ from each other.  7)The discussion of the “racial/ethnic group density” as it relates to HIV treatment outcomes are reported as they are in the included publications in the results section. This was purposeful so I can also provide discussion of how the results were reported in the discussion section. | 6)p.6-11, Lines 161-246  7)N/A |
| **Reviewer #1 - Discussion:**   8) As indicated above, constructs such as SES and “race/ethnicity densities” are taken as a given, without a nuanced discussion of what these mean. This needs to be improved. 9) Several sweeping sentences are delivered (i.e., Lines 317/318) that make generalizations regarding particular population subgroups without references or careful theory-driven discussions. | 8) More discussion has been detailed for the SES and racial/ethnicity densities constructs.  9)The sentences referenced on the previous version of the manuscript on Lines 317/318 have been conceptualized with more detail relating to the concept of density. | 8) p.12, Lines 261-264 and p.13, Lines 290-293 respectively  9)p.14, Lines 299-304 |
| **Reviewer #1 - Conclusion:**   10) The conclusions go beyond the scope of the review, talking about mechanisms, etc. I would focus the end to what the analysis actually found. | I have included a sentence that ties the conclusion to the scope of the review. However, I did leave the comment on research methods because it is something that affected the synthesis of the review. | 10)p.16-17, Lines 268-271 |

In summary, we believe that the revisions and clarifications made in response to the reviewers’ comments have strengthened the manuscript. We are grateful for the constructive feedback which has helped in improving the quality of our work.

We kindly request that you consider our revised manuscript for publication in PLOS Global Public Health. Please do not hesitate to contact us if you need any further information or clarification.

Yours sincerely,

Linda Jepkoech Kimaru, MPH

On behalf of all co-authors.
