# Supplementary material for: Neighborhood characteristics and HIV treatment outcomes: A scoping review
Source: PLOS Glob Public Health. 2024 Feb 13;4(2):e0002870. doi: 10.1371/journal.pgph.0002870 (PMC10863897; doi:10.1371/journal.pgph.0002870)
Supplement: Attachment — Submitted filename: LKimaru_PLOS GH_Rebuttal Letter_2.docx [file pgph.0002870.s004.docx]

University of Arizona

Mel and Enid Zuckerman College of Public Health

Department of Health Promotions Science

1295 N Martin Ave, Tucson, AZ 85724

P: +1 6024751517

E: [lkimaru@arizona.edu](mailto:lkimaru@arizona.edu)

The Staff Editor

Jianhong Zhou

PLOS Global Public Health

5 September 2023

**Re: Manuscript ID PGPH-D-23-00460 titled "** **Neighborhood characteristics and HIV treatment outcomes: A scoping review"**

I would like to thank you and the reviewers for the time and effort spent in evaluating our manuscript. We also thank you for considering our manuscript, and the opportunity to revise following peer review.

We are appreciative of the vital comments raised by the editor and have provided a revised manuscript. We have carefully addressed each of the concerns raised by the editor and made necessary revisions to the manuscript. Please find below a point-by-point response to the comments.

| **Editor’s Comment** | **Author’s Response** | **Page(s)- p;**  **Line(s)- L or supporting information document** |
| --- | --- | --- |
| **Journal Requirements**   1) Please ensure that you have addressed all items recommended in the PRISMA checklist. 2) and include the PRISMA flowchart as Figure 1. | 1) All the items in the PRISMA-ScR checklist have been addressed, please refer to supporting information – Lkimaru_PRISMA-ScR-Checklist pdf. 2) The conceptual model for the review is indicated as Figure 1 because it is the one that first appears and the PRISMA flow chart as Figure 2 because it appears second. This follows the [submission guidelines](https://journals.plos.org/globalpublichealth/s/submission-guidelines#loc-introduction) for the journal that states “*Cite figures in ascending numeric order at first appearance in the manuscript file*.” | 1) S1 File – PRISMA ScR Checklist pdf. 2) S1 Fig 1; S2 Fig 2 |
| 3) **The scoping review focused on the association of neighborhood characteristics and HIV treatment outcomes. However, in the introduction, the manuscript focuses on the lack of information on community factors and its relationship with HIV treatment outcomes. Then the manuscript talks about neighborhood characteristics. It is important to show how neighborhood characteristics links with community factors (through studies/)** 4) **and what are neighborhood characteristics are (with definitions and examples, and references).** | 3) In the introduction page 4-5 (lines 69-70) we specify that the community level examines settings such as neighborhoods. We also provided citation for the sentence. 4) In the introduction on page 5 (lines 71-73) we highlight the focus the neighborhood and provide a definition with citation. | 3) page 4-5 (lines 69-70) 4) page 5 (lines 71-73) |
| 5) **There are lack of definitions and references for key concepts in the manuscript. For example, the definition of HIV outcomes and neighbourhood characteristics need to be provided with references. How HIV outcomes are measured?** 6) **References are needed on neighbourhood characteristics definition, similarly, citation is needed: line 93-95 “The literature describes neighborhood characteristics in many ways using the tools, indicators, and datasets to characterize a neighborhood’s socioeconomic status (SES), deprivation index, disorder status, economic disadvantage, and overall environment”** | 5) HIV treatment outcomes are defined and measured in two places in the manuscript with references. In the introduction on page 4 (lines 56-57). In the methods section on page 8 (lines 147-154). Neighborhood characteristics definitions are introduced in page 5 (lines 89-91) with references. Then further detailed definitions with definitions and examples on SI Table 1. We decided on placing these definitions in a table format because it would be easier for an audience to read, given that it is a lot of text and would disrupt the flow. 6) Each of the neighborhood characteristics mentioned in the quoted text has an added citation. Thier definitions are detailed on S1 Table 1. This is because there is so much text that it would disrupt the flow. Therefore, we opted to put all the definitions on a table together with how they impact health. To ensure the reader knows to look at the table, we have added the following sentence “S1 Table 1 provides detailed definitions of these concepts and how they may impact health.” | 5) page 4 (lines 56-57); page 8 (lines 147-154); S1 Table 1 6) S1 Table 1 |
| 7) **Importantly, there is a lack of justification why scoping review approach is used to address the review questions.** 8) **The authors decided to use scoping review approach to address the review questions on assessing the influence of the association (Line 25) or examine the association (Line 89). Please use the terms and objectives consistently throughout the manuscripts.  As the authors use Arskey and O'Malley framework, how this framework could help to do review on the impact/ association of neighborhood characteristics and HIV treatment outcomes. It is important that the review specifies why scoping review is an appropriate approach, particularly why and how it can “critically examine the associations” (line 103) as no assessment of methodological quality nor formal data systhesis undertaken. Please clarify.** | 7) The protocol of the scoping review provided more information on why we used a scoping review approach to our research question. We do realize that we should be explicit in the main review as the protocol has not been published and therefore, we have updated the method section to that effect in page 6 (lines 104-116). 8) I agree that there was poor word choice when we used “critically examine”. The review itself summarized the scope of research on the topic. Therefore, we have re-worded the objective to be more appropriate and consistent with the review and the protocol. In the abstract (Page 2, Line 25-26), The wording has been modified to reflect the work on the review which was to summarize the scope of research available on the topic area. Also in the introduction, the objective was updated on (Page 6, lines 98-100) | 7) Methods - page 6 (lines 104-116 8) Abstract - Page 2 (Line 25-26); Introduction - (Page 6, lines 98-100) |
| 9) In the introduction: further information/ with examples is needed on how community factors links with neighbour characteristics. 10) Please be consistence with influence and association in the introduction. 11) Data on 2022 on HIV should be updated. | 9) In the introduction page 4-5 (lines 69-70) we specify that the community level examines settings such as neighborhoods. We also provided citation for the sentence. In the introduction on page 5 (lines 71-73) we highlight the focus the neighborhood and provide a definition with citation. 10) Throughout the manuscript we have replaced/removed the word influence where it seemed inappropriate 11) HIV statistics data in the introduction has been updated on Page 4 (Lines 48-49,53) | 9) Introduction - page 4-5 (lines 69-70); page 5 (lines 71-73) 10) Throughout the manuscript 11) Introduction - Page 4 (Lines 48-49,53) |
| 12) It is recommended that the authors should present clearly in the manuscript whether there are similar reviews on the topics. | 12) In the introduction on page 5-6 (Lines 91-98) we discuss how existing reviews on the topic of neighborhood characteristics and HIV will differ from the one we are presenting. | 12) Introduction - page 5-6 (Lines 91-98) |
| 13) How neighbourhood characteriscs were extracted and why should be further detailed. | 13) In the methods – eligibility criteria section on page 8 (Lines 146-147), we highlight that we used neighborhood characteristic definitions based on that of S1 Table 1, which are definitions from the literature. Also, on the Data charting and reporting section. I have added a sentence on how the abstraction was conducted on page 8 (Lines 160-163) | 13) Methods - eligibility criteria section on page 8 (Lines 146-147); S1 Table 1; page 8 (Lines 160-163) |
| 14) Search strategy: There is no limit on date of search, however, as HIV has been around since 1990s. Furthermore, the studies included in the scoping review only from 2006. Therefore, lower date limit for searches should be reconsidered. 15) Line 109: Databases’s platforms should be provided (Ovid, for instance). | 14) Our strategy had no lower date limit, on Page 7 (Line 119) we indicated that we searched from inception which means everything prior to our upper limit date. The studies that we ended up including met our inclusion criteria. 15) On Page 7 (Lines 119-120) we all listed the databases we used for our search with the aid from our Medical Librarian. We did not search on Ovid. | 14) Page 7 (Line 119) 15) Page 7 (Lines 119-120) |
| 16) Implications for research and limitations (on using scoping review to address the review question on associations) should be provided. | 16) In the limitation section on Page 17 (Lines 355-358) we included limitations and implications for research in using the scoping review methodology | 16) Page 17 (Lines 355-358) |

In summary, we believe that the revisions and clarifications made in response to the reviewers’ comments have strengthened the manuscript. We are grateful for the constructive feedback which has helped in improving the quality of our work.

We kindly request that you consider our revised manuscript for publication in PLOS Global Public Health. Please do not hesitate to contact us if you need any further information or clarification.

Yours sincerely,

Linda Jepkoech Kimaru, MPH

On behalf of all co-authors.
