# Supplementary material for: Neighborhood characteristics and HIV treatment outcomes: A scoping review
Source: PLOS Glob Public Health. 2024 Feb 13;4(2):e0002870. doi: 10.1371/journal.pgph.0002870 (PMC10863897; doi:10.1371/journal.pgph.0002870)
Supplement: Attachment — Submitted filename: LKimaru_PLOS GH_Rebuttal Letter_3.3.docx [file pgph.0002870.s005.docx]

University of Arizona

Mel and Enid Zuckerman College of Public Health

Department of Health Promotions Science

1295 N Martin Ave, Tucson, AZ 85724

P: +1 6024751517

E: [lkimaru@arizona.edu](mailto:lkimaru@arizona.edu)

The Academic Editor

Hien Thi HO, PhD, MD

PLOS Global Public Health

16 November 2023

**Re: Manuscript ID PGPH-D-23-00460R2 titled "** **Neighborhood characteristics and HIV treatment outcomes: A scoping review"**

I would like to thank you and the reviewers for the time and effort spent in evaluating our manuscript. We also thank you for considering our manuscript, and the opportunity to revise following peer review.

We are appreciative of the vital comments raised by the editor and have provided a revised manuscript. We have carefully addressed each of the concerns raised by the editor and made the required revisions to the manuscript. Please find below a point-by-point response to the comments.

| **Editor’s Comment** | **Author’s Response** | **Page(s)- p; Line(s)- L** |
| --- | --- | --- |
| 1. **Journal Requirements:** Please review your reference list to ensure that it is complete and correct. If you have cited papers that have been retracted, please include the rationale for doing so in the manuscript text or remove these references and replace them with relevant current references. Any changes to the reference list should be mentioned in the rebuttal letter that accompanies your revised manuscript. If you need to cite a retracted article, indicate the article’s retracted status in the References list and also include a citation and full reference for the retraction notice. | Each reference has been reviewed for completeness, correctness, and retraction status. Minor citation format issues identified have all been corrected. We confirm that no retracted articles were cited in the manuscript. Please review the changes below this letter. | N/A |
| **Journal Requirements**   2. Please ensure that you have addressed all items recommended in the PRISMA checklist. 3. and include the PRISMA flowchart as Figure 1. 4. Please amend the order of the figures, as the PRISMA flowchart has to be Figure 1 for systematic reviews and not the supplementary figures. You may move the other figure to later in the manuscript or to the supplemental information. | All the items in the PRISMA-ScR checklist have been addressed, please refer to supporting information – Lkimaru_PRISMA-ScR-Checklist pdf.  The PRISMA flowchart is now Figure 1, and the conceptual framework has been moved to supplemental information. We hope this meets the requirements. | S1 File – PRISMA ScR Checklist pdf.  Fig 1; S1 Conceptual Framework |
| 5. Please check the previous comment 15, platform of searching of databases should be provided, as searching different platforms for the same database could yield different studies. | After consulting the medical librarian, we have updated the verbiage on the searched database. We have included the host databases in brackets [e.g Embase (Elsevier)]. However, we did not include PubMed because it is a stand-alone database via the National Library of Medicine at the NIH. We hope this meets your expectations. | Abstract: p2; Line 27-28  Methods: p9; Line 126-129 |
| 6. Please consider revising lower date limit search as in comment 14 (of the previous responses to comment), the review focuses on HIV treatment outcomes, and HIV has been around since more than 30 years, therefore, searching from inception of the database is not incorrect but not necessary. | Thank you for this, we reviewed this again, and in response, we have noted in the manuscript, the start of each database searched, defined by the earliest publications. | Methods: p9; Line 126-129 |
| 7. Please provide explanation for the use Mendeley not EndNote later in the review process. The review used EndNote, Rayyan, and Mendeley software, which seems to be redundant. | We used EndNote for deduplication and then Rayyan QCRI for screening the publications according to the inclusion and exclusion criteria. We have updated the manuscript to remove Mendeley as that was just used for citation of the manuscript.  We also corrected the version date, as the librarian indicates the use of a later version then. | Pg 10, line 142-144 |
| 8. The review use the framework as provided in Fig.1. Further details / explanation how the framework is used to guide the review should be provided. | We thank the reviewers for this comment. We have provided an explanation of the framework and rationale for its use to guide the review process. | Pg 4-5; Line 66-79 |

In summary, we believe the revisions and clarifications made in response to the reviewers’ comments have strengthened the manuscript. We are grateful for the constructive feedback which has helped in improving the quality of our work.

We kindly request that you consider our revised manuscript for publication in PLOS Global Public Health. Please do not hesitate to contact us if you need any further information or clarification.

Yours sincerely,

Linda Jepkoech Kimaru, MPH

On behalf of all co-authors.

| **Notes** | **Before changes** | **After changes** |
| --- | --- | --- |
| We removed the reference as the webpage does not exist anymore, we felt the other four references provide sufficient evidence on the impacts of ART on improved health, and life expectancy, and reduced transmission. Therefore we did not feel the need to replace the reference with something else. | Centers for Disease Control and Prevention (CDC). How to Optimize HIV Treatment [Internet]. p. 1–8. Available from: https://www.cdc.gov/hiv/clinicians/treatment/treatment-clinicians.html |  |
| Updated title from 'FAST-TRACK: Ending the AIDS Epidemic by 2030' to 'Understanding FAST-TRACK: Accelerating Action to End the AIDS Epidemic by 2030' | UNAIDS. FAST-TRACK: Ending the AIDS Epidemic by 2030 [Internet]. 2015. p. 283. Available from: https://www.unaids.org/sites/default/files/media_asset/201506_JC2743_Understanding_FastTrack_en.pdf | UNAIDS. Understanding FAST-TRACK: Accelerating Action to End the AIDS Epidemic by 2030 [Internet]. 2015. p. 283. Available from: https://www.unaids.org/sites/default/files/media_asset/201506_JC2743_Understanding_FastTrack_en.pdf |
| Missing 3 authors - added - James A. Mercy, Anthony B. Zwi and Rafael Lozano | Dahlberg L, Krug E. Violence: a global public health problem [Internet]. World Report on Violence and Health. [cited 2021 Mar 7]. Available from: http://apps.who.int/iris/bitstream/handle/10665/42495/9241545615_eng.pdf | Dahlberg L, Krug E, Mercy JA, Zwi AB, Lozano R. Violence: a global public health problem [Internet]. World Report on Violence and Health. [cited 2021 Mar 7]. Available from: http://apps.who.int/iris/bitstream/handle/10665/42495/9241545615_eng.pdf |
| web address added | Roux AVD. Investigating neighborhood and area effects on health. Am J Public Health. 2001;91(11):1783–9. | Diez Roux A V. Investigating Neighborhood and Area Effects on Health. Am J Public Health [Internet]. 2001 Nov;91(11):1783–9. Available from: https://ajph.aphapublications.org/doi/full/10.2105/AJPH.91.11.1783 |
| web address added | Prentice JC. Neighborhood effects on primary care access in Los Angeles. Soc Sci Med. 2006 Mar;62(5):1291–303. | Prentice JC. Neighborhood effects on primary care access in Los Angeles. Soc Sci Med [Internet]. 2006 Mar;62(5):1291–303. Available from: http://www.ncbi.nlm.nih.gov/pubmed/16129534 |
| web address added | Frank LD, Iroz-Elardo N, MacLeod KE, Hong A. Pathways from built environment to health: A conceptual framework linking behavior and exposure-based impacts. J Transp Heal. 2019 Mar;12:319–35. | Frank LD, Iroz-Elardo N, MacLeod KE, Hong A. Pathways from built environment to health: A conceptual framework linking behavior and exposure-based impacts. J Transp Heal [Internet]. 2019 Mar;12:319–35. Available from: https://linkinghub.elsevier.com/retrieve/pii/S2214140518303360 |
| website rectified | Bauermeister JA, Zimmerman MA, Caldwell CH. Neighborhood Disadvantage and Changes in Condom Use among African American Adolescents. J Urban Heal [Internet]. 2011 Feb 14;88(1):66–83. Available from: http://ezproxy.library.arizona.edu/login?url=https://search.ebscohost.com/login.aspx?direct=true&db=rzh&AN=104824551&site=ehost-live | Bauermeister JA, Zimmerman MA, Caldwell CH. Neighborhood Disadvantage and Changes in Condom Use among African American Adolescents. J Urban Heal [Internet]. 2011 Feb 14;88(1):66–83. Available from: http://link.springer.com/10.1007/s11524-010-9506-9 |
| web address added | Latkin CA, Curry AD, Hua W, Davey MA. Direct and indirect associations of neighborhood disorder with drug use and high-risk sexual partners. Am J Prev Med. 2007/08/19. 2007;32(6 Suppl):S234-41. | Latkin CA, Curry AD, Hua W, Davey MA. Direct and Indirect Associations of Neighborhood Disorder With Drug Use and High-Risk Sexual Partners. Am J Prev Med [Internet]. 2007/08/19. 2007 Jun;32(6):S234–41. Available from: https://linkinghub.elsevier.com/retrieve/pii/S0749379707001110 |
| web address added | Parrado EA, Flippen C. Community attachment, neighborhood context, and sex worker use among Hispanic migrants in Durham, North Carolina, USA. Soc Sci Med. 2010/02/04. 2010;70(7):1059–69. | Parrado EA, Flippen C. Community attachment, neighborhood context, and sex worker use among Hispanic migrants in Durham, North Carolina, USA. Soc Sci Med [Internet]. 2010/02/04. 2010 Apr;70(7):1059–69. Available from: https://linkinghub.elsevier.com/retrieve/pii/S0277953610000389 |
| website rectified | Maas B, Fairbairn N, Kerr T, Li K, Montaner JSG, Wood E. Neighborhood and HIV infection among IDU: Place of residence independently predicts HIV infection among a cohort of injection drug users. Heal Place [Internet]. 2007;13(2):432–9. Available from: https://www.embase.com/search/results?subaction=viewrecord&id=L46136069&from=export http://dx.doi.org/10.1016/j.healthplace.2006.05.005 | Maas B, Fairbairn N, Kerr T, Li K, Montaner JSG, Wood E. Neighborhood and HIV infection among IDU: place of residence independently predicts HIV infection among a cohort of injection drug users. Health Place [Internet]. 2007 Jun;13(2):432–9. Available from: http://www.ncbi.nlm.nih.gov/pubmed/16798055 |
| web address added | Friedman SR, Mateu-Gelabert P, Curtis R, Maslow C, Bolyard M, Sandoval M, et al. Social capital or networks, negotiations, and norms? A neighborhood case study. Am J Prev Med. 2007/08/19. 2007;32(6 Suppl):S160-70. | Friedman SR, Mateu-Gelabert P, Curtis R, Maslow C, Bolyard M, Sandoval M, et al. Social Capital or Networks, Negotiations, and Norms? A Neighborhood Case Study. Am J Prev Med [Internet]. 2007/08/19. 2007 Jun;32(6):S160–70. Available from: https://linkinghub.elsevier.com/retrieve/pii/S0749379707000621 |
| web address added | Howe CJ, Siegel H, Dulin-Keita A. Neighborhood Environments and Sexual Risk Behaviors for HIV Infection Among U.S. Women: A Systematic Review. AIDS Behav. 2017;21(12):3353–65. | Howe CJ, Siegel H, Dulin-Keita A. Neighborhood Environments and Sexual Risk Behaviors for HIV Infection Among U.S. Women: A Systematic Review. AIDS Behav [Internet]. 2017 Dec 19;21(12):3353–65. Available from: http://link.springer.com/10.1007/s10461-017-1771-0 |
| web address added | Sampson RJ, Raudenbush SW, Earls F. Neighborhoods and violent crime: A multilevel study of collective efficacy. Science (80- ). 1997;277(5328):918–24. | Sampson RJ, Raudenbush SW, Earls F. Neighborhoods and Violent Crime: A Multilevel Study of Collective Efficacy. Science (80- ) [Internet]. 1997 Aug 15;277(5328):918–24. Available from: https://www.science.org/doi/10.1126/science.277.5328.918 |
| web address added | Arksey H, O’Malley L. Scoping studies: Towards a methodological framework. Int J Soc Res Methodol Theory Pract. 2005;8(1):19–32. | Arksey H, O’Malley L. Scoping studies: towards a methodological framework. Int J Soc Res Methodol [Internet]. 2005 Feb;8(1):19–32. Available from: http://www.tandfonline.com/doi/abs/10.1080/1364557032000119616 |
| web address added | World Health Organisation, Unicef, Unaids. A Guide on Indicators for Monitoring and Reporting on the Health Sector Response to HIV / AIDS. 2011. | WHO, UNICEF, UNAIDS. A Guide on Indicators for Monitoring and Reporting on the Health Sector Response to HIV / AIDS [Internet]. 2011. Available from: https://www.unaids.org/sites/default/files/media_asset/20111130_UA_Report_en_1.pdf |
| corrected webpage reference - removed Vol.1 Hong Kong Journal of Dermatology and Venerology | CDC. HIV treatment as prevention [Internet]. Vol. 21, Hong Kong Journal of Dermatology and Venereology. 2019. Available from: https://www.cdc.gov/hiv/risk/art/index.html | Centers for Disease Control and Prevention (CDC). HIV treatment as prevention [Internet]. Available from: https://www.cdc.gov/hiv/risk/art/index.html |
| We kept the article as the erratum on the article was on not related to the findings of the article. The erratum in th article indicated " *ErratumIn the article by Joy et al, appearing in the Journal of Acquired Immune Deficiency Syndromes, Vol. 47, No. 4, pp. 500–505, entitled ‘‘Impact of Neighborhood-Level Socioeconomic Status on HIV Disease Progression in a Universal Health Care Setting,’’ author Eric Druyts misrepresented himself by reporting to have a degree that he did not complete. Therefore,Eric Drutys should not have an MSc after his name, as is currently listed on pp. 500 of the article.*" the erratum citation - (2010). Impact of Neighborhood-Level Socioeconomic Status on HIV Disease Progression in a Universal Health Care Setting: Erratum. JAIDS Journal of Acquired Immune Deficiency Syndromes, 53 (3), 424. doi: 10.1097/QAI.0b013e3181d79b2f | Joy R, Druyts EF, Brandson EK, Lima VD, Rustad CA, Zhang W, et al. Impact of Neighborhood-Level Socioeconomic Status on HIV Disease Progression in a Universal Health Care Setting. JAIDS J Acquir Immune Defic Syndr [Internet]. 2008/01/17. 2008 Apr 1;47(4):500–5. Available from: https://journals.lww.com/00126334-200804010-00013 | Joy R, Druyts EF, Brandson EK, Lima VD, Rustad CA, Zhang W, et al. Impact of Neighborhood-Level Socioeconomic Status on HIV Disease Progression in a Universal Health Care Setting. JAIDS J Acquir Immune Defic Syndr [Internet]. 2008/01/17. 2008 Apr 1;47(4):500–5. Available from: https://journals.lww.com/00126334-200804010-00013 |
| website rectified | Jefferson KA, Kersanske LS, Wolfe ME, Braunstein SL, Haardörfer R, Jarlais DC Des, et al. Place-Based Predictors of HIV Viral Suppression and Durable Suppression Among Men Who Have Sex With Men in New York City. AIDS Behav [Internet]. 2017 Oct 23;21(10):2987–99. Available from: http://ezproxy.library.arizona.edu/login?url=https://search.ebscohost.com/login.aspx?direct=true&db=psyh&AN=2017-28176-001&site=ehost-live kevin.a.jefferson@emory.edu | Jefferson KA, Kersanske LS, Wolfe ME, Braunstein SL, Haardörfer R, Jarlais DC Des, et al. Place-Based Predictors of HIV Viral Suppression and Durable Suppression Among Men Who Have Sex With Men in New York City. AIDS Behav [Internet]. 2017 Oct 23;21(10):2987–99. Available from: http://link.springer.com/10.1007/s10461-017-1810-x |
| website rectified | Chen Y-N, Coker D, Kramer MR, Johnson BA, Wall KM, Ordóñez CE, et al. The Impacts of Residential Location on the Risk of HIV Virologic Failure Among ART Users in Durban, South Africa. AIDS Behav [Internet]. 2019 Sep 2;23(9):2558–75. Available from: http://ezproxy.library.arizona.edu/login?url=https://search.ebscohost.com/login.aspx?direct=true&db=rzh&AN=138866004&site=ehost-live | Chen Y-N, Coker D, Kramer MR, Johnson BA, Wall KM, Ordóñez CE, et al. The Impacts of Residential Location on the Risk of HIV Virologic Failure Among ART Users in Durban, South Africa. AIDS Behav [Internet]. 2019 Sep 2;23(9):2558–75. Available from: http://link.springer.com/10.1007/s10461-019-02523-7 |
| website rectified | Wiewel EW, Borrell LN, Maroko AR, Jones HE, Torian L V, Udeagu C-C. Neighborhood social cohesion and viral suppression after HIV diagnosis. J Health Psychol [Internet]. 2021 Mar 5;26(3):477–85. Available from: http://ezproxy.library.arizona.edu/login?url=https://search.ebscohost.com/login.aspx?direct=true&db=rzh&AN=148800404&site=ehost-live | Wiewel EW, Borrell LN, Maroko AR, Jones HE, Torian L V, Udeagu C-C. Neighborhood social cohesion and viral suppression after HIV diagnosis. J Health Psychol [Internet]. 2021 Mar 5;26(3):477–85. Available from: http://journals.sagepub.com/doi/10.1177/1359105318810088 |
| web address added | Caleb-Adepoju SO, Dawit R, Gbadamosi SO, Sheehan DM, Fennie KP, Ladner RA, et al. Factors Associated with Viral Suppression Among Racial/Ethnic Minority Women in the Miami-Dade County Ryan White Program, 2017. AIDS Res Hum Retroviruses. 2021 Sep 1;37(9):631–41. | Caleb-Adepoju SO, Dawit R, Gbadamosi SO, Sheehan DM, Fennie KP, Ladner RA, et al. Factors Associated with Viral Suppression Among Racial/Ethnic Minority Women in the Miami-Dade County Ryan White Program, 2017. AIDS Res Hum Retroviruses [Internet]. 2021 Sep 1;37(9):631–41. Available from: http://www.ncbi.nlm.nih.gov/pubmed/34078113 |
| web address added | Dawit R, Trepka MJ, Duncan DT, Li T, Pires SF, Brock P, et al. Neighborhood Factors Associated with Racial/Ethnic Disparities in Achieving Sustained HIV Viral Suppression Among Miami-Dade County Ryan White Program Clients. AIDS Patient Care STDS. 2021 Oct 1;35(10):401–10. | Dawit R, Trepka MJ, Duncan DT, Li T, Pires SF, Brock P, et al. Neighborhood Factors Associated with Racial/Ethnic Disparities in Achieving Sustained HIV Viral Suppression Among Miami-Dade County Ryan White Program Clients. AIDS Patient Care STDS [Internet]. 2021 Oct 1;35(10):401–10. Available from: http://www.ncbi.nlm.nih.gov/pubmed/34623889 |
| web address added | López JD, Qiao Q, Presti RM, Hammer RA, Foraker RE. The impact of neighborhood socioeconomic status on retention in care and viral suppression among people living with HIV. AIDS Care. 2022 Nov 2;34(11):1383–9. | López JD, Qiao Q, Presti RM, Hammer RA, Foraker RE. The impact of neighborhood socioeconomic status on retention in care and viral suppression among people living with HIV. AIDS Care [Internet]. 2022 Nov 2;34(11):1383–9. Available from: http://www.ncbi.nlm.nih.gov/pubmed/35164602 |
| Web address added, we kept the article as the erratum is regarding a title misprint and we did not affect the findings. The note on the erratum indicates "*The original version of this article unfortunately contained a misprint for the title of Fig. 3, and it has been corrected with this erratum. The correct title is given below: Unadjusted logistic regression and adjusted multilevel logistic regression models of viral suppression.*" | Hovhannisyan L, Coelho LE, Velasque L, De Boni RB, Clark J, Cardoso SW, et al. Multilevel Analysis of Individual and Neighborhood Characteristics Associated with Viral Suppression Among Adults with HIV in Rio de Janeiro, Brazil. AIDS Behav. 2022 Mar 25;26(3):947–62. | Hovhannisyan L, Coelho LE, Velasque L, De Boni RB, Clark J, Cardoso SW, et al. Correction to: Multilevel Analysis of Individual and Neighborhood Characteristics Associated with Viral Suppression Among Adults with HIV in Rio de Janeiro, Brazil. AIDS Behav [Internet]. 2022 Mar 1;26(3):963–963. Available from: https://link.springer.com/10.1007/s10461-021-03528-x |
| web address added | Wright IA, Reid R, Shahid N, Ponce A, Nelson CM, Sanders J, et al. Neighborhood Characteristics, Intersectional Discrimination, Mental Health, and HIV Outcomes Among Black Women Living With HIV, Southeastern United States, 2019‒2020. Am J Public Health. 2022 Jun;112(S4):S433–43. | Wright IA, Reid R, Shahid N, Ponce A, Nelson CM, Sanders J, et al. Neighborhood Characteristics, Intersectional Discrimination, Mental Health, and HIV Outcomes Among Black Women Living With HIV, Southeastern United States, 2019‒2020. Am J Public Health [Internet]. 2022 Jun;112(S4):S433–43. Available from: http://www.ncbi.nlm.nih.gov/pubmed/35763751 |
| A full title of the article added | Goswami ND, Schmitz MM, Sanchez T, Dasgupta S, Sullivan P, Cooper H, et al. Understanding Local Spatial Variation Along the Care Continuum. JAIDS J Acquir Immune Defic Syndr [Internet]. 2015/12/03. 2016 May 1;72(1):65–72. Available from: https://journals.lww.com/00126334-201605010-00010 | Goswami ND, Schmitz MM, Sanchez T, Dasgupta S, Sullivan P, Cooper H, et al. Understanding Local Spatial Variation Along the Care Continuum: The Potential Impact of Transportation Vulnerability on HIV Linkage to Care and Viral Suppression in High-Poverty Areas, Atlanta, Georgia. J Acquir Immune Defic Syndr [Internet]. 2015/12/03. 2016 May 1;72(1):65–72. Available from: https://journals.lww.com/00126334-201605010-00010 |
| web address added | Øvrum A, Gustavsen GW, Rickertsen K. Age and socioeconomic inequalities in health: Examining the role of lifestyle choices. Adv Life Course Res. 2014 Mar;19:1–13. | Ovrum A, Gustavsen GW, Rickertsen K. Age and socioeconomic inequalities in health: examining the role of lifestyle choices. Adv Life Course Res [Internet]. 2014 Mar;19:1–13. Available from: http://www.ncbi.nlm.nih.gov/pubmed/24796874 |
| web address added | Mendenhall E, Kohrt BA, Norris SA, Ndetei D, Prabhakaran D. Non-communicable disease syndemics: poverty, depression, and diabetes among low-income populations. Lancet. 2017 Mar;389(10072):951–63. | Mendenhall E, Kohrt BA, Norris SA, Ndetei D, Prabhakaran D. Non-communicable disease syndemics: poverty, depression, and diabetes among low-income populations. Lancet (London, England) [Internet]. 2017 Mar 4;389(10072):951–63. Available from: http://www.ncbi.nlm.nih.gov/pubmed/28271846 |
| website rectified | Wilkes R, Iceland J. Hypersegregation in the twenty-first century. Demography [Internet]. 2004 Feb 1;41(1):23–36. Available from: https://read.dukeupress.edu/demography/article/41/1/23/170281/Hypersegregation-in-the-twenty-first-century | Wilkes R, Iceland J. Hypersegregation in the twenty-first century. Demography [Internet]. 2004 Feb 1;41(1):23–36. Available from: http://www.ncbi.nlm.nih.gov/pubmed/15074123 |
| website rectified | Hays RB, Rebochook GM, Kegeles SM. The Mpowerment Project: Community-building with young gay and bisexual men to prevent HIV. Am J Community Psychol [Internet]. 2003;31(3–4):301–12. Available from: http://ezproxy.library.arizona.edu/login?url=https://search.ebscohost.com/login.aspx?direct=true&db=psyh&AN=2003-05825-012&site=ehost-live skegeles@psg.ucsf.edu | Hays RB, Rebchook GM, Kegeles SM. The Mpowerment Project: community-building with young gay and bisexual men to prevent HIV1. Am J Community Psychol [Internet]. 2003 Jun;31(3–4):301–12. Available from: http://www.ncbi.nlm.nih.gov/pubmed/12866687 |
| web address added | Sørensen JFL. Rural–Urban Differences in Bonding and Bridging Social Capital. Reg Stud. 2016 Mar 3;50(3):391–410. | Sørensen JFL. Rural–Urban Differences in Bonding and Bridging Social Capital. Reg Stud [Internet]. 2016 Mar 3;50(3):391–410. Available from: http://www.tandfonline.com/doi/full/10.1080/00343404.2014.918945 |
| web address added | Avery EE, Hermsen JM, Kuhl DC. Toward a Better Understanding of Perceptions of Neighborhood Social Cohesion in Rural and Urban Places. Soc Indic Res. 2021 Sep 17;157(2):523–41. | Avery EE, Hermsen JM, Kuhl DC. Toward a Better Understanding of Perceptions of Neighborhood Social Cohesion in Rural and Urban Places. Soc Indic Res [Internet]. 2021 Sep 17;157(2):523–41. Available from: https://link.springer.com/10.1007/s11205-021-02664-0 |
| web address added | Moskowitz D, Vittinghoff E, Schmidt L. Reconsidering the Effects of Poverty and Social Support on Health: A 5-Year Longitudinal Test of the Stress-Buffering Hypothesis. J Urban Heal. 2013 Feb 5;90(1):175–84. | Moskowitz D, Vittinghoff E, Schmidt L. Reconsidering the effects of poverty and social support on health: a 5-year longitudinal test of the stress-buffering hypothesis. J Urban Health [Internet]. 2013 Feb 5;90(1):175–84. Available from: http://www.ncbi.nlm.nih.gov/pubmed/22948789 |
| Duplicate reference- removed (same as reference 25) | Prentice JC. Neighborhood effects on primary care access in Los Angeles. Soc Sci Med [Internet]. 2006 Mar;62(5):1291–303. Available from: https://linkinghub.elsevier.com/retrieve/pii/S0277953605004028 |  |
